# Identification of Putative Markers That Predict the In Vitro Senescence of Mesenchymal Progenitor Cells

**DOI:** 10.3390/cells10061301

**Published:** 2021-05-24

**Authors:** Eun-Young Shin, Yeo-Joon Yoon, Jeoung Eun Lee, Sung Han Shim, Gene Hong Park, Dong Ryul Lee

**Affiliations:** 1 Department of Biomedical Science, CHA University, Seongnam 13488, Gyunggi-do, Korea; eunyoungs0514@naver.com (E.-Y.S.); dbsduwns9899@naver.com (Y.-J.Y.); shshim@cha.ac.kr (S.H.S.); jinhpark96@naver.com (G.H.P.); 2 CHA Advanced Research Institute, CHA University, Seongnam 13488, Gyunggi-do, Korea; jel43@chamc.co.kr

**Keywords:** stem cell therapy, good manufacturing practice, proliferation, high-quality MPC, pluripotent stem-cell-derived mesenchymal progenitor cell

## Abstract

Mesenchymal progenitor cells (MPCs) are a promising cell source for regenerative medicine because of their immunomodulatory properties, anti-inflammatory molecule secretion, and replacement of damaged cells. Despite these advantages, heterogeneity in functional potential and limited proliferation capacity of MPCs, as well as the lack of suitable markers for product potency, hamper the development of large-scale manufacturing processes of MPCs. Therefore, there is a sustained need to develop highly proliferative and standardized MPCs in vitro and find suitable functional markers for measuring product potency. In this study, three lines of pluripotent stem cell (PSC)-derived MPCs with high proliferative ability were established and compared with bone-marrow-derived MPCs using proliferation assays and microarrays. A total of six genes were significantly overexpressed (>10-fold) in the highest proliferative MPC line (CHA-hNT5-MPCs) and validated by qRT-PCR. However, only two of the genes (MYOCD and ODZ2) demonstrated a significant correlation with MPC senescence in vitro. Our study provides new gene markers for predicting replicative senescence and the available quantity of MPCs but may also help to guide the development of new standard criteria for manufacturing.

## 1. Introduction

Mesenchymal progenitor cells (MPCs) are defined as adherent stromal cells that are multipotent and have a self-renewal potential in various tissues. MPCs can be obtained from bone marrow, adipose tissue, endometrium, dental tissue, placenta, umbilical cord, and Wharton’s jelly [1]. According to the International Society for Cellular Therapy (ISCT), minimal criteria must be met in order to characterize human MPCs. Firstly, cells must adhere to a plastic dish, and second, they must present specific surface antigens such as CD105, CD73, and CD90; however, they should not express hematopoietic markers, such as CD45, CD34, and human leukocyte antigen-DR (HLA-DR). Finally, they must have differentiation potential to adipocytes, osteoblasts, and chondroblasts [2].

MPCs can be homing to the injured site and not only replace damaged cells but also encourage tissue regeneration. Moreover, these cells can secrete various cytokines and growth factors that promote cell proliferation, increase cell viability, and confer immune tolerance [1,2]. Owing to these benefits, MPCs can be used as therapeutic agents. Hence, currently there are approximately 1015 registered trials evaluating the effect of MPCs on diverse diseases (clinicaltrials.gov, accessed on 20 May 2021).

Although MPCs have been widely used experimentally, they have limitations in clinical use. In order to achieve sufficient therapeutic effects, clinical applications require large quantities of cells and standardized manufacturing systems [3]. However, MPCs have donor heterogeneity and limited cell proliferation capacity in culture and are likely to vary in efficacy depending on culture conditions, such as cell density, passage, and growth factor supplementation [4,5,6,7]. Thus, the major challenge for MPC therapy is the development of a standardized, large-scale manufacturing protocol and the identification of proper markers for quality control. Moreover, MPCs from diverse tissues require an invasive and painful procedure for extraction that carries the risk of infection; therefore, alternative sources are needed.

Pluripotent stem cells (PSCs) can be supplied unlimitedly, had clear biological background and are useful for scale-up production [8]. Recently, pluripotent stem cell-derived MPCs (PSC-MPCs) have been used as alternative sources for tissue-derived MPCs due to their high proliferative potential and ease of standardization [8]. Several studies have shown that PSC-MPCs proliferate faster and have shorter population doubling (PD) times than bone-marrow-derived MPCs [9,10,11]. Similarly, we previously reported that somatic cell nuclear transfer (SCNT) and human embryonic stem cell (hESC)-derived MPCs have a higher proliferation capacity and shorter doubling time than human bone-marrow-derived MPCs (hBM-MPCs) in vitro [12]. Thus, it is important to understand the characteristics of high proliferative PSC-MPCs because they could be used as limitless and universal therapy. However, numerous studies have focused on the similarities and differences between tissue-derived MPCs and PSC-MPCs at the characteristic, genetic, and epigenetic levels, rather than on the factors behind differences in growth potency [13,14,15]. If we identify the overexpressed factors present in highly proliferative MPCs in vitro, they could be used for predicting quality and proliferation capacity while using MPCs in the clinic.

To address this, we first generated three different cell lines of PSC-MPCs to obtain a highly proliferative and well-maintained MPC line. We then cultured all types of MPCs until they reached growth arrest to verify the highest proliferative line. Finally, we compared transcriptomic changes among MPCs to identify new markers that can predict growth potential and senescence in vitro.

## 2. Materials and Methods

### 2.1. Culture of Human PSCs

Three different PSC lines were used in this study: conventional ESCs (CHA-hES15, Korea Stem Cell Registry No. hES12010028) and SCNT-PSCs (CHA-hNT5, Korea Stem Cell Registry No. hES22014015 and CHA-hNT8, Korea Stem Cell Registry No. hES22015003). All PSCs were cultured on mitotically inactivated mouse embryonic fibroblasts (MEFs, CF1 strain, Jackson laboratory, Los Gotos, CA, USA) in ESC medium consisting of DMEM/F12 medium (Gibco, Franklin Lakes, NJ, USA) supplemented with 20% knockout serum replacement (Gibco), 0.1 mM beta-mercaptoethanol (Gibco), 1% non-essential amino acids (NEAA, Gibco), and 4 ng/mL recombinant human basic fibroblast growth factor (bFGF, Invitrogen, CA, USA). ESC medium was changed daily, and cells were passaged every 4 days.

### 2.2. Differentiation of Human PSCs into MPCs

Human PSCs were detached by a mechanical method using a Pasteur pipette (Corning) and cultured in a Petri dish (Corning) for embryoid body (EB) formation. The EB medium consisted of hESC medium without bFGF and was supplemented with 1 µM SB431542 (Sigma-Aldrich). Fourteen days after formation, EBs were attached to culture dishes and outgrowth cells (OGs) were maintained in DMEM/low glucose (Hyclone, Logan, UT, USA) supplemented with 10% fetal bovine serum (FBS, Gibco-BRL), 0.1 mM beta-mercaptoethanol, 1× NEAA, and 1× penicillin-streptomycin (P/S, Gibco). Sixteen days after EB attachment, OGs were subcultured in MPC medium that consisted of DMEM/high glucose (Hyclone) supplemented with 10% FBS, 0.1 mM beta-mercaptoethanol, 1× NEAA, and 1× P/S.

### 2.3. Flow Cytometry

Detached cells were briefly resuspended in 200 µL of FACS buffer (1% FBS in PBS) and incubated with antibodies for 30 min. We used CD29-APC, CD44-APC, CD90-APC, CD105-APC as MPC markers, and CD34-APC, CD45-APC, SSEA4-APC, TRA-1-60-PE (Miltenyi Biotec, Bergisch Gladbach, Germany) as hematopoietic and stem cell markers. Cells were washed twice with FACS buffer and analyzed using a FACSCalibur cytometer (BD Biosciences, San Jose, CA, USA).

### 2.4. Mesodermal Lineage Differentiation

The differentiation capacity of MPCs into adipocytes, osteocytes, and chondrocytes has been described in a previous report [15]. In brief, for adipogenic and osteogenic differentiation, MPCs were seeded at 5 × 10^4^ cells/well in 0.1% gelatin-coated 4-well tissue culture dishes in MPC medium. After 1 d, culture medium was changed to adipogenic medium (StemPro^®^ Adipogenesis Differentiation Kit, Gibco-BRL) or osteogenic medium (StemPro^®^ Osteogenesis Differentiation Kit, Gibco-BRL). After 3 weeks, samples were fixed with 4% paraformaldehyde solution (PFA) and stained with Oil Red O solution (HC World, Woodstock, MD, USA) to stain the lipid droplets in the cells. To confirm the intracellular calcium deposits in osteocytes, samples were stained with alizarin red solution (Sigma-Aldrich).

For chondrogenic differentiation, MPCs were seeded at 5 × 10^5^ cells/well into a 15 mL conical tube (BD Falcon, Lexington, TN, USA) and cultured with StemPro^®^ Chondrogenesis Differentiation Kit (Gibco-BRL). After 4 weeks, samples were fixed with 4% PFA and embedded in paraffin. Chondrocytes in the sectioned samples were identified by staining the cell surface alkaline phosphatase with Alcian blue solution (Cat. IW3000, IHC World) for 10 min and counter-staining with nuclear fast solution (IHC world).

### 2.5. Cell Proliferation Assay

For the analysis of cell proliferation during long-term culture, cells were maintained in MPC medium and seeded at 2–1.5 × 10^5^ cells/well into a 6-well plate. Cells were counted, and the proliferation curve and doubling time between cell passages were evaluated as previously described [12].

### 2.6. Microarray Analysis

Total RNA of each MPC was extracted using TRIzol reagent (Takara) according to the manufacturer’s protocol. To synthesize RNA, 50 ng of total RNA was used for amplification. The RNA was labeled using the biotin labeling method. Hybridization was performed using the Human Gene ST 2.0 Array (Affymetrix, Santa Clara, CA, USA). Array washing and staining were performed using Fluidics 450. Microarray data were obtained using a GeneChip scanner 3000 7G (Affymetrix), and the whole microarray process was performed at Bio-core (Bio-core, Seoul, Korea). Significantly overexpressed genes were sorted by Log2 fold change values compared with BM-MPCs (Log2 fold change >1, *p* < 0.05). Gene Ontology (GO) and Venn diagrams were analyzed using Enrich R and Venny.

### 2.7. Reverse Transcription Polymerase Chain Reaction (RT-PCR) and Quantitative Real-Time PCR (qRT-PCR)

Isolation of total RNA and cDNA synthesis was performed as previously described [12]. For conventional PCR, PCR reactions (20 µL) contained 1 µL of each primer (10 pM), 2 µL of 10× Taq reaction buffer, 1 µL of 10 mM dNTP mix, and 0.2 µL of Taq DNA polymerase (5 U/μL, SolGent Co., Ltd., Daejeon, Korea). Each product was amplified for 30 cycles at 60 °C using appropriate primers. For real-time PCR, SYBR green PCR master mixes (Enzynomics) were used, and all target genes were calculated using the comparative CT method. All genes were normalized to β-actin and the primer sequences used in this study are listed in Table 1.

## 3. Results

### 3.1. Differentiation of MPCs from PSCs

**Figure 1:**
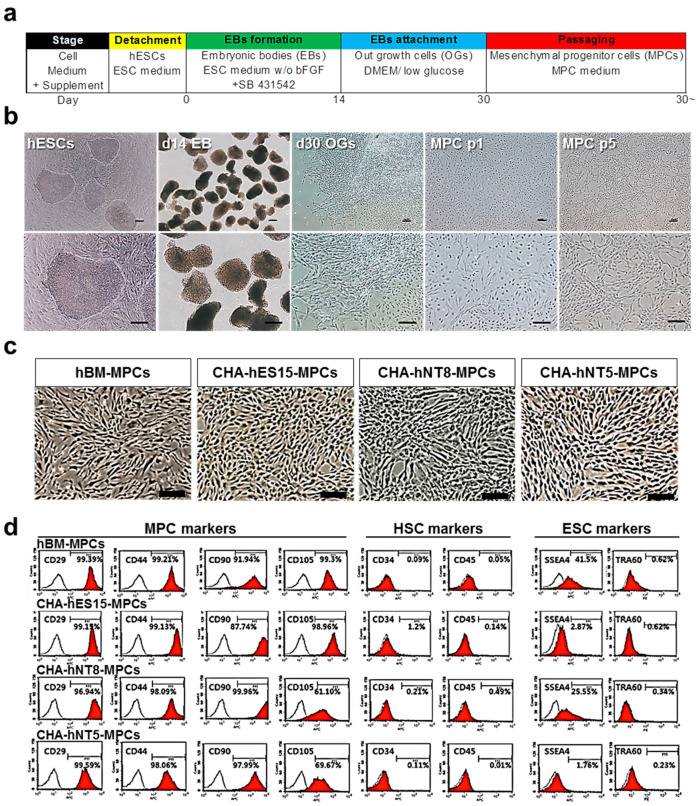
Differentiation of hPSC into MPCs. (**a**) Experimental protocol for differentiation of pluripotent stem cells into mesenchymal progenitor cells (PSC-MPCs). Scale bars, 100 µm. (**b**) Representative images of cell morphology in each differentiation stage. Scale bars, 100 µm. (**c**) Representative phase contrast images of hBM-MPCs and hPSC-MPCs lines (CHA-hES15, CHA-NT8, CHA-NT5) at passage 5. All MPCs showed fibroblastic morphology. Scale bars = 200 µm. (**d**) FACS analysis showed that all MPCs expressed positive for MPCs markers (CD29, CD44, CD90, CD105) but negative for HSC markers (CD34, CD45) and ESC markers (SSEA4, TRA-1-60). Abbreviations: hESCs, human embryonic stem cells; MPCs, mesenchymal progenitor cells; HSC, hematopoietic stem cell; hBM-MPCs, human bone-marrow-derived mesenchymal progenitor cells.

Several studies reported that PSC-derived MPCs had higher proliferation capacity than adult tissue-derived MPCs, so we established PSC-MPC lines from three different donor-derived PSCs to obtain high proliferation rates and overcome the inbuilt replication limit of MPCs [8]. In addition, we used human BM-MPCs as positive controls for further analysis, as they are widely considered as a standard for adult tissue-derived MPCs. According to previous reports, MPCs can be generated from a mesodermal origin. Thus, we attempted to induce the mesodermal lineage during EB formation using SB431542, which is known as a TGF-beta/nodal/activin signaling inhibitor that can induce mesoderm lineage (Figure 1a) [16,17]. To obtain the MPCs from EBs, mesoderm-rich EBs were attached to the plate, and outgrowth cells were collected by trypsinization at 16 days after attachment. Thereafter, the cells were selected by detachment times to obtain a homogenous population and further cultured until passage 5 (Figure 1b). All types of MPCs generated from different PSC cell lines exhibited a fibroblastic morphology analogous to that of BM-MPCs (Figure 1c).

### 3.2. PSC-Derived MPCs Show Typical MSC Markers and Have Differentiation Capacity

**Figure 2:**
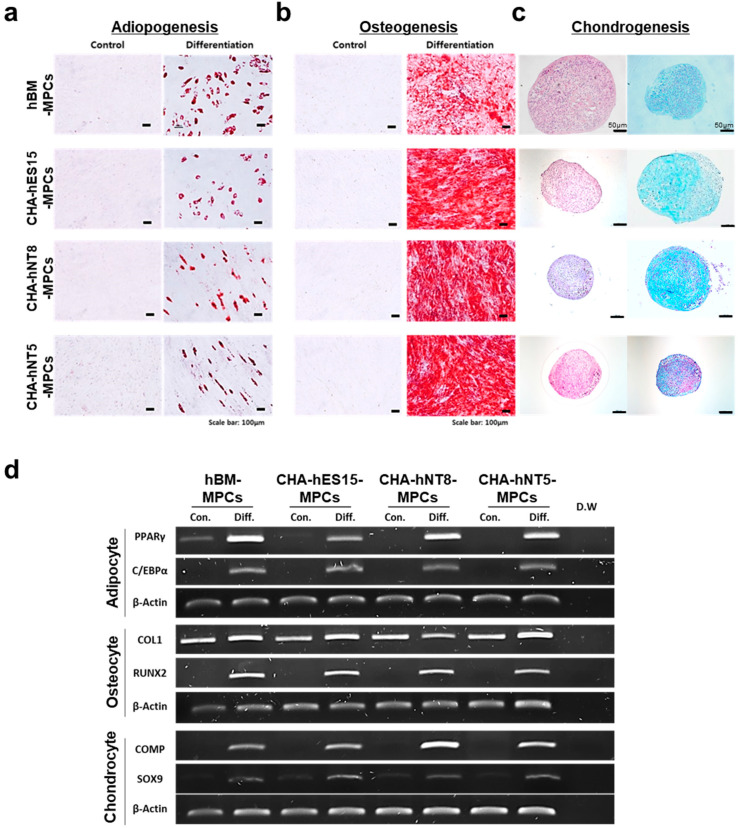
Multipotent differentiation of MPCs. To confirm their multipotent potential, all MPCs were tested to evaluate differentiation potentials for (**a**) adipogenesis—scale bars, 100 µm; (**b**) osteogenesis—scale bars, 100 µm; (**c**) chondrogenesis—scale bars, 50 µm and 100 µm. (**d**) RT-PCR was performed to confirm the expression of positive genes for adipocytes, osteocytes, and chondrocytes in each cell line. Abbreviations: MPCs, mesenchymal progenitor cells; Con, control; Diff, differentiation; D.W, distilled water.

According to the ISCT guidelines, MPCs must express specific surface antigens and have the ability to exhibit multipotent differentiation potential [2]. Therefore, to further characterize MPCs, cell surface markers were analyzed using a flow cytometer. Figure 1d shows that all PSC-derived MPCs were positive for MPC markers, such as CD29, CD44, CD90, and CD105, but negative for hematopoietic markers (CD34 and CD45). Furthermore, all types of MPCs were able to differentiate into adipocytes, osteoblasts, and chondrocytes (Figure 2a–d).

### 3.3. PSC-MPCs Showed a Robust Growth Potential

**Figure 3:**
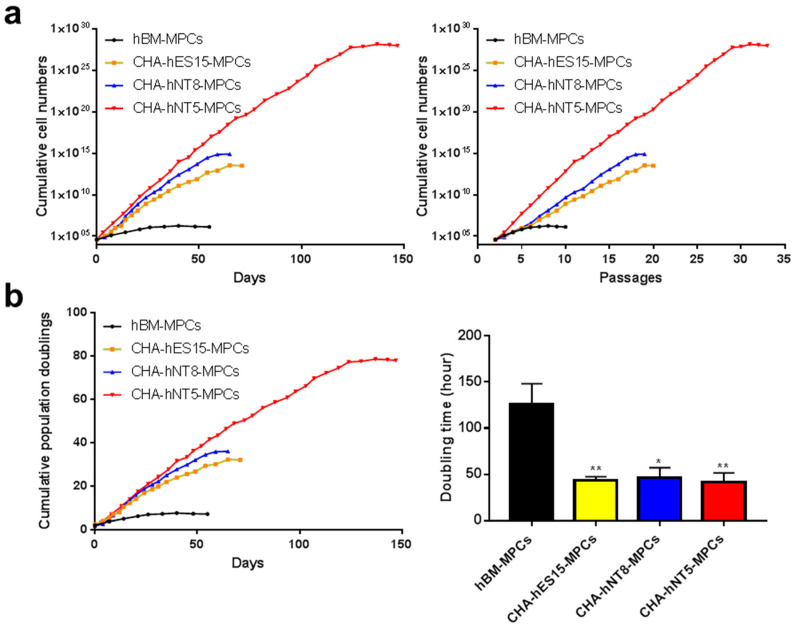
Growth kinetics of MPCs. Cumulative population doubling levels (CPDL) from hBM-MPCs, CHA-hES15-MPCs, CHA-hNT8-MPCs and CHA-hNT5-MPCs were recorded and compared. (**a**) Cumulative cell number of MPCs was recorded. (**b**) Cumulative population doubling and doubling times of MPCs were calculated. Bars represent mean ± SE. Significant difference is analyzed by student’s *t* test, where: * *p* < 0.05, ** *p* < 0.01. Abbreviations: SE, standard error.

To evaluate the proliferative level of all types of MPCs in vitro, we maintained the cells until they underwent replicative senescence. All PSC-MPCs (CHA-hES15-MPCs, CHA-hNT8-MPCs, and CHA-hNT5-MPCs) showed exponential growth compared to hBM-MPCs. hBM-MPCs gradually decreased their cell division after eight passages, resulting in cell senescence (Figure 3a). As shown in Figure 3b, hBM-MPCs had a replication limit within 7.53 PDs over 50 days, but MPCs had 26.60–36.15 PDs over the same period. Furthermore, the doubling time of all PSC-MPCs was significantly shorter than that of hBM-MPCs (2.87-fold), suggesting that PSC-MPCs can rapidly and sufficiently expand for therapy over short periods. Unexpectedly, CHA-hNT5-MPCs showed more robust proliferation than any other MPCs. Therefore, we hypothesized that the identification of genes that are overexpressed in CHA-hNT5-MPCs compared with other MPCs could be used to establish the MPCs with high proliferative potential.

### 3.4. Identification of Differentially Expressed Genes in High Proliferative MPCs

**Figure 4:**
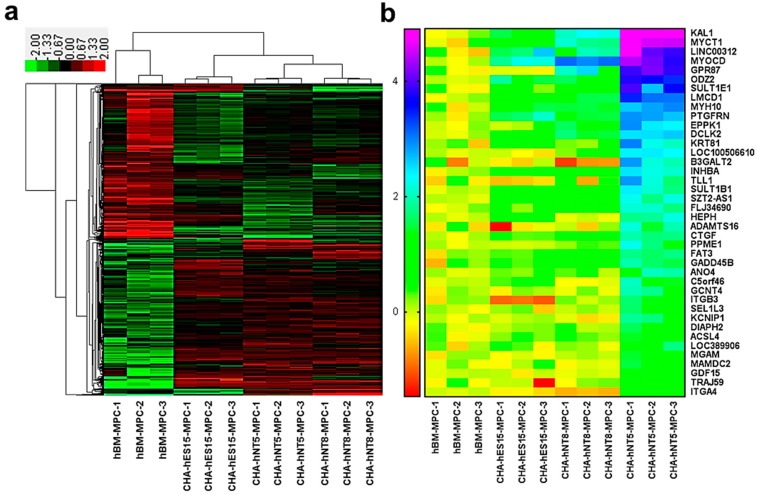
Transcriptome analysis of gene expression profiling by microarray. (**a**) Heatmap of the differently expressed genes (DEGs) of hBM-MPCs and corresponding PSC-MPCs. Hierarchical clustering clearly separated hBM-MPCs and PSC-MPCs. (**b**) Heatmap presentation of enriched genes in CHA-hNT5-MPCs compared with other MPCs. (Only common genes are presented, fold changes >2 and adjusted *p* < 0.05.).

To identify genes that were differentially expressed in CHA-hNT5-MPCs, which had high proliferative ability, RNAs were isolated from the same passage of hBM-MPCs, CHA-hES15-MPCs, CHA-hNT8-MPC, and CHA-hNT5-MPCs. RNA was extracted and then analyzed via microarray, and a total of 2392 transcriptomes were identified as significantly differentially expressed genes (DEG, Figure 4a). Next, we measured the overexpressed transcripts in PSC-MPCs against hBM-MPCs because all PSC-MPCs showed a robust proliferation index in continuous culture compared to hBM-MPCs.

**Figure 5:**
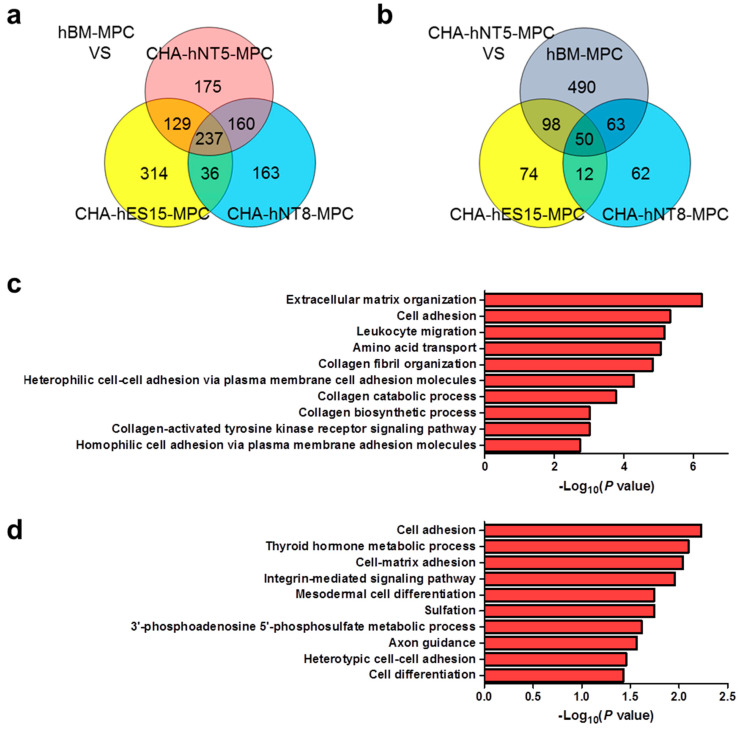
Venn diagram and functional annotation analysis of microarray data. (**a**) Venn diagram showing the number and overlap of overexpressed genes in PSC-MPCs compared with hBM-MPCs. A total of 237 transcriptomes were identified. (**b**) Venn diagrams showing the 50 transcriptomes that are up-regulated in CHA-hNT5-MPCs against other MPCs. (**c**) Enriched biological processes of significantly up-regulated genes in the PSC-MPCs compared with hBM-MPCs are shown as bar diagram with –log10 (*p* value). (**d**) Biological process GO terms of up-regulated genes in CHA-hNT5-MPCs were presented as bar diagrams with –log10 (*p* value). Abbreviations: PSC-MPCs, pluripotent stem cell-derived mesenchymal progenitor cells; hBM-MPCs, human bone-marrow-derived mesenchymal progenitor cells; GO, gene ontology.

Among the 2392 DEGs, the expression of 237 transcripts was greater than 2-fold higher in PSC-MPCs than in hBM-MPCs (Figure 5a). Functional annotation analysis of these transcriptomes revealed that they were involved in extracellular matrix organization, cell adhesion, and the biological process of collagen formation (Figure 5c). Finally, since CHA-hNT5-MPCs had the most robust exponential growth kinetics compared to that of other MPCs, we made a comparison between CHA-hNT5-MPCs and other MPCs. In total, 50 transcriptomes (Table 2) were overexpressed in CHA-hNT5-MPCs and annotated to cell adhesion and related to metabolic processes (Figure 5b,d). Based on these results, we speculated that cell adhesion and extracellular matrix related genes might be related to the proliferation capacity of MPCs and their senescence in vitro.

### 3.5. Evaluation of Selected Genes on Different Passages of MPCs

**Figure 6:**
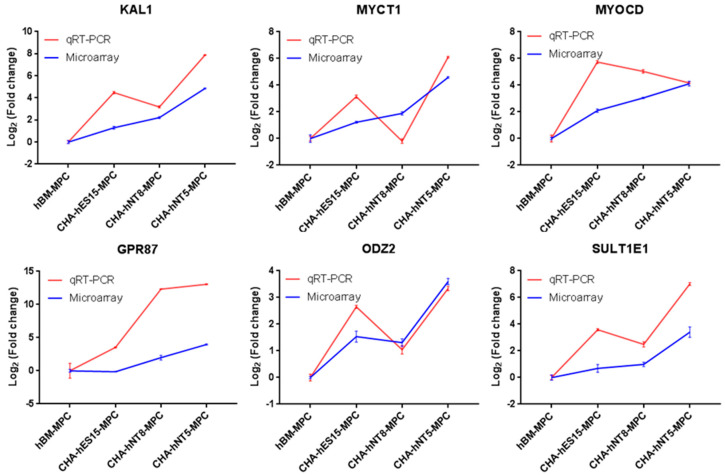
Validation of selected genes identified by microarray. Blue lines represent microarray results and red lines represent qRT-PCR results. The relative expression levels of genes were normalized with the internal control gene GAPDH. Cell types are on the *x*-axis, and the log2 fold change in transcription relative to hBM-MPCs is on the *y*-axis. Error bar represents standard error (SE). Abbreviations: qRT-PCR, quantitative real time polymerase chain reaction.

To validate the reliability of the microarray results, the top six common genes from among the 50 differentially expressed transcripts were selected and validated by qRT-PCR. Consistent with the microarray data, KAL1, MYCT1, MYOCD, GPR87, ODZ2, and SULT1E1 levels were significantly high in the CHA-hNT5-MPCs (Figure 6). The expression of these six genes was greater than 10-fold higher in CHA-hNT5-MPCs than in hBM-MPCs, which might be associated with their proliferation potential and senescence in vitro because CHA-hNT5-MPCs had the highest growth rate.

**Figure 7:**
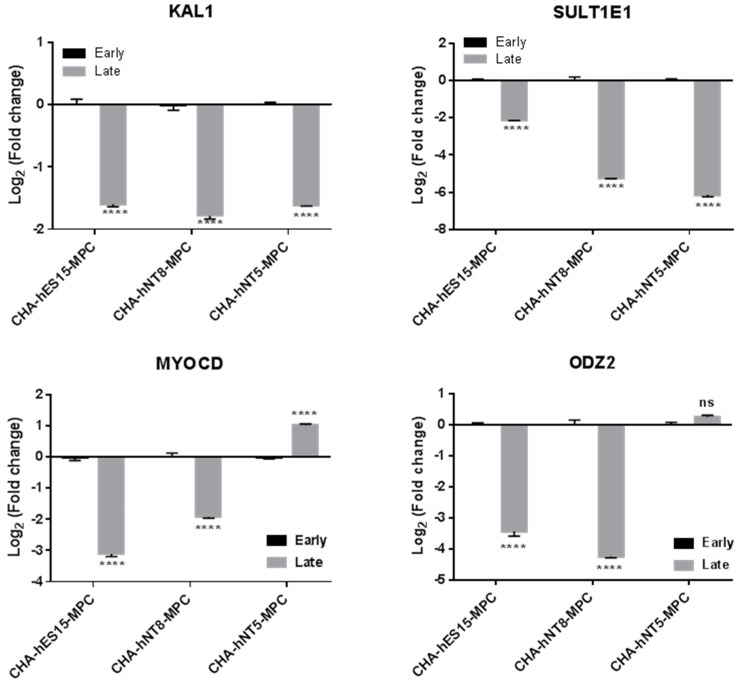
Selected gene expression for the different passages of PSC-MPCs. qRT-PCR was used to evaluate gene expression in cultured PSC-MPCs at early (P5) and late (P18) passage. Log2 fold change in each MPC calculated relative to expression of early (P5) passage and normalized to the GAPDH is shown. Bars represent mean ± SE. Significant difference between passages is analyzed by student’s *t* test, where: ****, *p* < 0.0001. Abbreviations: Early, early passage (P5); Late, late passage (P18); P, passage; SE, standard error.

To further assess the expression differences during replicative senescence, we evaluated the expression of these genes in the early and late passages of MPCs (Figure 7). As shown in Figure 3, CHA-hES15-MPCs and CHA-hNT8-MPCs underwent replicative senescence in passage 18; therefore, we chose passage 18 as the late passage. In contrast, CHA-hNT5-MPCs showed a high growth rate in the same passages. Thus, we assumed that if the expression of genes that changed by passage was not altered in CHA-hNT5-MPCs during passaging, that gene could be used as a putative marker for predicting replicative aging. The expression of KAL1, MYOCD, ODZ2, and SULT1E1 in late passages (p18) of CHA-hES15-MPCs and CHA-hNT8-MPCs was significantly lower than that of early passage (p5). However, the expression of MYOCD and ODZ2 in CHA-hNT5-MPCs was similar in early and late passages. These data suggest that these two genes might play important roles in the proliferation of late-stage MPCs.

## 4. Discussion

In the present study, we analyzed the characteristics of three different PSC-MPCs, which have a higher proliferative capacity than tissue-derived MPCs, and identified putative markers related to their proliferation and senescence in vitro. Specifically, we hypothesized that there is clearly heterogeneity among PSC-MPCs from PSC lines with different genetic background and tried to find genes related to hyper-proliferation from this condition. In particular, MYOCD and ODZ2 demonstrated a significant correlation with replicative senescence of MPCs in vitro from microarray analysis. Our findings provide new insight into predicting the proliferative capacity of MPCs and help for MPC industry.

MPCs have the ability to modulate immune cells, reduce inflammation, regenerate tissue, induce apoptosis, and improve angiogenesis. As a result of these properties, MPCs have been used to treat various diseases. However, the manufacturing of MPCs has various challenges, such as limited cell number, lack of standardized manufacturing processes, and markers for evaluating their quality and potency.

The production of MPCs for clinical use must comply with good manufacturing practice (GMP), which includes the expansion of cells to achieve a sufficient quantity. The successful MPC industry process also requires maintaining high quality and potency of cells, along with cost-effectiveness and reliability of the process. However, there are no standardized methods for manufacturing MPCs because of their heterogeneity and biological complexity. This might be addressed by quality-by-design (QbD) principles, which enable translating laboratory experiments into industrial processes [18]. According to QbD, the strategy for manufacturing begins with quality target product profile (QTPP), defined as an explanation of the desired product quality characteristics such as identity, purity, and potency of MPCs. Quality attributes (CQAs) that can affect the safety and efficacy of a product must be identified to ensure the desired quality. Finally, the critical process parameter (CPP) that can impact a CQA should be monitored to guarantee that the process generates the desired quality. For example, CPPs for in vitro expansion of MPCs can be associated with cell density, age, medium, supplement, pH, temperature, and dissolved oxygen [19]; therefore, these parameters must be consistently monitored and controlled during the manufacturing of MPCs. Nonetheless, as mentioned above, tissue-derived MPCs still have the biological intricacy and heterogeneity from derived tissue sources or donors that can predispose each MPC to unique CQAs; hence, the equivalent CPPs must be distinguished case by case [20].

In the present study, all PSC-MPC lines had a higher proliferative potential than hBM-MPC lines and were able to be generated in a stable condition, irrespective of donor (Figure 1, Figure 2 and Figure 3). Furthermore, we confirmed that MPCs could proliferate well under simple and standardized culture medium conditions. These results suggest that the quality and potency of MPCs could be standardized through our differentiation method and, thus, achieve a large number of cells for therapeutic efficacy. This proliferative advantage may enable PSC-MPCs to be an alternative source for tissue-derived MPC products. MPCs for therapeutic applications require at least 1–2 × 10^6^ cells per kilogram (kg), which means that at least 1 × 10^8^ cells are needed for a single dose [20]. The number of isolated MPCs from tissue aspiration was dependent on their source, but the average number of cells was 0.4 × 10^6^ MPCs per donation. Thus, an expansion factor of at least 250-fold is required. As shown in Figure 3a, if 4 × 10^4^ cells were cultured at the start, we were able to harvest approximately 1.85 × 10^6^ cells from hBM-MPCs and more than 8 × 10^12^ cells from PSC-MPCs. Based on this result, the number of hBM-MPCs that can be obtained from 0.4 × 10^6^ cells, that is the average number of cells per donation, is 1.85 × 10^7^. Thus, this quantity is approximately 5.41 times less than the minimum amount required for a single dose, while PSC-MPC can help to obtain a sufficient number of cells for therapeutic application. In addition, PSC-MPCs had 3.53-fold more PDs than those in hBM-MPC over the same period, suggesting that using PSC-MPCs can dramatically reduce the culture periods and be easily scaled up to supply sufficient cells for therapy.

To guarantee the desired quality and quantity of MPC products, replicative senescence-related changes must be monitored during long-term culture. There are some methods to track changes in long-term culture, such as monitoring the CPDL, SA-β-galactosidase, and telomere length. However, there is still a lack of suitable markers for cellular aging and for cross-validated genes in different MPCs. Thus, it is difficult to predict the status of the senescent stage, which can affect the expected total cell numbers for harvest. To solve this, we compared gene expression changes in MPCs that have different proliferative capacities, to identify the proper markers for predicting growth potential. As shown in Table 2, CHA-hNT5-MPC, which is the highest proliferative cell, overexpressed 50 transcriptomes compared to other MPCs. The top six common genes were selected and validated by qRT-PCR and compared to the early and late passages of each MPC. We found that only four genes (KAL1, MYOCD, ODZ2, and SULT1E1) were significantly decreased in the late passages (p18) of CHA-hES15-MPC and CHA-hNT8-MPC (Figure 7). However, MYOCD and ODZ2 maintained similar expression levels in the CHA-hNT5-MPCs. As most cells underwent senescence at passage 18 while CHA-hNT5-MPCs still had a high proliferative potential, this result supports that these genes (MYOCD, ODZ2) were maintained well in CHA-hNT5-MPCs and thus might be related to replicative senescence.

Myocardin (MYOCD) plays an important role in smooth muscle differentiation and cardiomyocyte survival [21,22]. MYOCD can regulate the expression of actin and cytoskeleton through interaction with the serum response factor (SRF), which can regulate the stiffness of vascular smooth muscle cells by inducing actin polymerization [23]. ODZ2, also known as Teneurin Transmembrane Protein 2 (TENM2), is a cell surface adhesion protein that plays a critical role in embryogenesis [24]. ODZ2 works as a linker protein that can connect the cytoskeleton to other cells or the extracellular matrix (ECM) while simultaneously having signal capabilities [25]. In particular, the intracellular domain of ODZ2 acts as an anchor to bind the actomyosin cytoskeleton and regulate the strengthening of cell–cell adhesion [26]. The common factor shared by these two genes is that they are involved in cytoskeleton regulation. Recently, several studies have reported that ECM proteins can maintain the proliferation and differentiation potential of MPCs [27]. ECM stiffness can regulate cell spreading, stress fiber formation, focal adhesion formation, and intracellular stiffening, which can stimulate the proliferation and motility of cells [28]. Our present data also revealed that ECM organization, cell adhesion, and collagen fibril organization related GO terms were enriched in PSC-MPCs, which have higher proliferative potency than hBM-MPCs (Figure 5c,d). These results suggest that maintaining the expression of cytoskeleton-related genes such as MYOCD and ODZ2 may sustain the proliferation of MPCs at late passages. However, our study only confirmed the gene expression; thus, further studies are required to confirm the protein expression and roles of these genes in replicative senescence of MPCs.

## 5. Conclusions

In conclusion, we successfully generated high proliferative MPCs from three different cell lines of PSCs and found promising putative markers for predicting the replicative senescence of MPCs by comparing the gene expressions of each cell line using microarray analysis. MYOCD and ODZ2 were identified as significantly correlated with proliferation capacity of PSC-MPCs, and these markers might be used as a new critical process parameter in the MPC industry.

## Figures and Tables

**Table 1:** Primers for quantitative RT-PCR.

Gene	Primer Sequence (5′ -> 3′)	
*β-ACTIN*	Forward	TGAAGTGTGACGTGGACATC
	Reverse	GGAGGAGCAATGATCTTGAT
*PPARγ*	Forward	TGTCTCATAATGCCATCAGGTTTG
	Reverse	GATAACGATGGTGATTTGTCTGTT
*C/EBPα*	Forward	GCAAACTCACCGCTCCAATG
	Reverse	TTAGGTTCCAAGCCCCAAGTC
*COL-1*	Forward	AGAACATCACCTACCACTGC
	Reverse	ATGTCCAAAGGTGCAATATC
*RUNX2*	Forward	CCGCACGACAACCGCACCAT
	Reverse	CGCTCCGGCCCACAAATCTC
*COMP*	Forward	AACGCTGAAGTCACGCTCAC
	Reverse	GGTAGCCAAAGATGAAGCCC
*SOX9*	Forward	TTCATGAAGATGACCGACGA
	Reverse	CACACCATGAAGGCGTTCAT
*GAPDH*	Forward	AGAAGGCTGGGGCTCATTTG
	Reverse	AGGGGCCATCCACAGTCTTC
*GPR87*	Forward	CTACCTTGTCTGGTAGGGGAGATG
	Reverse	TCAGCATAGGTTATTCCTGGTTTG
*MYCT1*	Forward	GCCAGAAAACTTTTGGGAGGA
	Reverse	ATCCAGTTCTGTTGAGGCCG
*SULT1E1*	Forward	AAAGAGGGTGATGTGGAA
	Reverse	AAATGAGGCAGGAAGAAG
*KAL1*	Forward	AGCGGAGAAAGACTACGGATGG
	Reverse	GGACACCTTTGCACTCTTCAGC
*ODZ2*	Forward	CCTCTCGAAATGTGACCAGCATC
	Reverse	GCGGTAGATTCTCCTGCTGTTG
*MYOCD*	Forward	CCACCTATGGACTCAGCCTAC
	Reverse	CTCAGTGGCGTTGAAGAAGAG

**Table 2:** Upregulated genes in high proliferative CHA-hNT5-MPCs compared with other MPCs, as analyzed using microarray; only genes that have fold change (CHA-hNT5-MPC/other MPCs) ≥2 are presented. Only differences that were statistically significant at *p* < 0.05 as determined by *t*-test are reported. Log2 fold change of genes that are exclusively expressed in CHA-hNT5-MPCs was presented relative to hBM-MPCs.

mRNA Accession	Gene Symbol	Gene Description	Gene Accession	Log2 Fold Change (Relative to hBM-MPCs)		
				CHA-hES15-MPCs	CHA-hNT8-MPCs	CHA-hNT5-MPCs
NM_001302777	-	-	-	0.973	0.253	4.896
NM_001143981	KAL1	Kallmann syndrome 1 sequence	NM_000216	1.299	2.210	4.853
NR_026776	MYCT1	myc target 1	NM_025107	1.222	1.887	4.588
XR_937373	LINC00312	long intergenic non-protein coding RNA 312	NR_024065	1.954	1.690	4.227
NONHSAT052653	MYOCD	myocardin	NM_001146312	2.098	3.046	4.112
NM_001011655	GPR87	G protein-coupled receptor 87	NM_023915	−0.140	1.986	3.969
-	-	-	-	2.151	1.539	3.842
NM_001102562	ODZ2	odz, odd Oz/ten-m homolog 2 (Drosophila)	NM_001122679	1.534	1.310	3.599
NM_001271156	-	-	-	1.990	0.109	3.461
NM_001128843	SULT1E1	sulfotransferase family 1E, estrogen-preferring, member 1	NM_005420	0.684	0.989	3.406
NM_001308394	LMCD1	LIM and cysteine-rich domains 1	ENST00000157600	0.972	1.376	3.189
NM_002196	-	-	-	1.290	0.161	3.097
BC037342	MYH10	myosin, heavy chain 10, non-muscle	NM_001256012	1.303	1.462	2.796
NM_017709	PTGFRN	prostaglandin F2 receptor negative regulator	NM_020440	1.779	1.433	2.793
ENST00000391302	-	-	-	1.157	1.432	2.733
NR_121212	EPPK1	epiplakin 1	ENST00000525985	0.160	1.127	2.583
NM_001127266	DCLK2	doublecortin-like kinase 2	NM_001040261	0.644	1.540	2.542
ENST00000549438	KRT81	keratin 81	NM_002281	0.987	0.356	2.427
NM_001256155	LOC100506610	uncharacterized LOC100506610	ENST00000446964	−0.176	0.449	2.400
NR_104625	-	-	-	−0.263	0.693	2.363
NM_005558	B3GALT2	UDP-Gal:betaGlcNAc beta 1,3-galactosyltransferase, polypeptide 2	ENST00000367434	−0.224	−0.865	2.338
NM_001084	INHBA	inhibin, beta A	ENST00000242208	0.554	1.050	2.272
XR_241687	TLL1	tolloid-like 1	NM_012464	−0.448	0.162	2.234
NONHSAT098134	SULT1B1	sulfotransferase family, cytosolic, 1B, member 1	ENST00000310613	0.545	0.829	2.102
NONHSAT002938	SZT2-AS1	SZT2 antisense RNA 1 (non-protein coding)	ENST00000396885	0.782	0.995	2.076
ENST00000432942	-	-	-	0.214	−0.136	2.008
NM_004295	FLJ34690	uncharacterized protein FLJ34690	NR_034145	0.525	0.906	1.991
NM_001017991	HEPH	hephaestin	ENST00000519389	0.922	−0.077	1.948
NM_001143974	-	-	-	−0.001	0.336	1.944
NM_020726	ADAMTS16	ADAM metallopeptidase with thrombospondin type 1 motif, 16	NM_139056	−0.633	−0.084	1.856
ENST00000375094	CTGF	connective tissue growth factor	ENST00000367976	0.346	0.607	1.806
GENSCAN00000011063	PPME1	protein phosphatase methylesterase 1	ENST00000328257	−0.014	0.263	1.738
NM_000829	FAT3	FAT tumor suppressor homolog 3 (Drosophila)	NM_001008781	0.369	0.634	1.726
NM_021915	GADD45B	growth arrest and DNA-damage-inducible, beta	NM_015675	0.485	0.596	1.703
NM_001101421	ANO4	anoctamin 4	NM_178826	0.273	0.362	1.659
NM_003804	C5orf46	chromosome 5 open reading frame 46	NM_206966	0.496	−0.157	1.591
NM_001009185	GCNT4	glucosaminyl (N-acetyl) transferase 4, core 2	NM_016591	0.024	−0.001	1.581
NM_203425	ITGB3	integrin, beta 3 (platelet glycoprotein IIIa, antigen CD61)	NM_000212	−0.963	0.126	1.470
NM_006168	SEL1L3	sel-1 suppressor of lin-12-like 3 (C. elegans)	NM_015187	−0.007	0.069	1.426
NM_004061	KCNIP1	Kv channel interacting protein 1	NM_001034837	0.050	−0.247	1.388
NM_001412	DIAPH2	diaphanous homolog 2 (Drosophila)	NM_007309	0.150	0.166	1.329
NR_001553	ACSL4	acyl-CoA synthetase long-chain family member 4	NM_022977	0.313	0.283	1.328
NONHSAT138126	LOC389906	zinc finger protein 839 pseudogene	NR_034031	0.220	0.073	1.263
ENST00000365229	-	-	-	−0.130	−0.083	1.220
NM_030661	MGAM	maltase-glucoamylase (alpha-glucosidase)	ENST00000549489	−0.150	0.068	1.219
XM_011519004	MAMDC2	MAM domain containing 2	NM_153267	0.118	−0.130	1.174
NM_001300974	GDF15	growth differentiation factor 15	NM_004864	−0.074	−0.060	1.132
ENST00000362934	TRAJ59	T cell receptor alpha joining 59 (non-functional)	ENST00000390480	−0.452	0.088	1.124
XR_427224	ITGA4	integrin, alpha 4 (antigen CD49D, alpha 4 subunit of VLA-4 receptor)	NM_000885	−0.292	−0.566	1.029
NM_031273	-	-	-	−0.206	−0.849	1.019

## Data Availability

All data are included in the paper.
